# A Family-Centered, Multidisciplinary Clinic for Early Diagnosis of Neurodevelopmental Impairment and Cerebral Palsy in China—A Pilot Observation

**DOI:** 10.3389/fped.2022.840190

**Published:** 2022-03-17

**Authors:** Hai-Bo Huang, Man Joe Watt, Matthew Hicks, Qian-Shen Zhang, Fang Lin, Xue-Qing Wan, Chun-Bong Chow, Po-Yin Cheung

**Affiliations:** ^1^Department of Pediatrics, University of Hong Kong-Shenzhen Hospital, Shenzhen, China; ^2^Department of Pediatrics, Glenrose Rehabilitation Hospital, University of Alberta, Edmonton, AB, Canada; ^3^Department of Physical Medicine and Rehabilitation, Glenrose Rehabilitation Hospital, University of Alberta, Edmonton, AB, Canada; ^4^Department of Pediatrics, University of Alberta, Edmonton, AB, Canada; ^5^Department of Physical Medicine and Rehabilitation, University of Hong Kong-Shenzhen Hospital, Shenzhen, China

**Keywords:** prematurity, cerebral palsy, neurodevelopmental impairment (NDI), multidisciplinary (care or team), early diagnosis, neurodevelopment

## Abstract

**Background:**

Comprehensive multidisciplinary assessment of neurodevelopmental outcomes of high-risk neonates may have significant challenges in low- and middle-income countries, in addition to socio-cultural barriers. We aimed to compare the time to diagnosis of neurodevelopmental impairment (NDI) and cerebral palsy (CP) in preterm neonates (<29 weeks) at a multidisciplinary assessment and care (MDAC) clinic with that of a conventional high-risk infant follow-up clinic in China.

**Methods:**

All eligible surviving very preterm neonates born at <29 weeks gestation at the University of Hong Kong–Shenzhen Hospital between January 2015 and December 2019 were followed up in conventional (2015–2017) and MDAC (2018–2020) clinics up to 2 years corrected age with clinical demographic information collected in a prospective database. The MDAC team used standardized developmental assessments. The rates and timing of diagnosing NDI and CP in two epochs were compared.

**Results:**

The rates of NDI and CP were not different in two epochs [NDI: 12 (50%) vs. 12 (41%); CP: 3 (12%) vs. 2 (7%) of 24 and 29 surviving infants assessed in conventional and MDAC clinics, respectively]. Infants in the MDAC clinic were diagnosed with NDI and CP earlier than those in the pre-MDAC epoch (6 vs. 14 months corrected age, respectively, *P* < 0.05).

**Conclusion:**

High-risk preterm neonates can be followed more effectively in a family-centered, child-friendly multidisciplinary clinic, leading to an earlier diagnosis of NDI and CP. Early counseling and interventions could be implemented accordingly.

## Introduction

Although the survival of very preterm infants continues to improve, extremely preterm birth remains a leading cause of neonatal death, short-term morbidity, and neurodevelopmental sequelae in early childhood (1). Among the neurodevelopmental sequelae during early childhood, neurodevelopmental impairment (NDI) is a composite outcome that includes significant motor, cognitive, and neurosensory impairments (2). Cerebral palsy (CP) is a group of permanent disorders of the development of movement and posture, causing activity limitation that is attributed to non-progressive disturbances that occurred in the developing fetal or infant brain (3). In a National Institute of Child Health and Human Development study of extremely preterm infants (≤ 27 weeks gestation) between 2011 and 2015, 12% had CP and 16–34% had moderate-to-severe NDI between 18 and 26 months adjusted age (4). It was estimated that 3.5–14.9% of extremely preterm infants developed severe NDI in the Canadian Neonatal Follow-up Network (2). In China, there is limited information or large cohort studies regarding the neurodevelopmental outcome in extremely preterm infants.

A study from the Global low- and middle-income countries CP register recruited 2,664 children from Bangladesh, Nepal, Indonesia, and Ghana between January 2015 and May 2019 and showed that the median age of diagnosing CP was 3 years (5). In Vietnam, using a surveillance system modeled on the Pediatric Active Enhanced Disease Surveillance system in Australia, CP was diagnosed at a mean age of 1 year and 8 months (6). In China, CP was usually diagnosed at 1–2 years of age (7). However, two recent studies showed that early diagnosis of CP in the first year of life in high-risk infant follow-up clinics is both feasible and practical (8, 9). Further, many infants with NDI and CP respond well to interventions in early childhood when brain plasticity is at its greatest (10). Therefore, early diagnosis of NDI and CP could be crucial to influence the outcomes of these children and their families. Based on moderate-quality evidence, Novak et al. (11) advocated that the diagnosis of CP can and should be made as soon as possible so that (1) the infant can receive diagnostic-specific early intervention and surveillance to optimize neuroplasticity and prevent complications and (2) the parents can receive psychological and financial support. From the family-centered care perspective, most parents of children with CP want earlier diagnosis and do not want information withheld. They value honest and accurate information and research-driven diagnosis and treatment (12).

We aimed to evaluate the feasibility of implementing an “arena assessment” model by a multidisciplinary team and its timing in the diagnosis of CP and NDI in infants <29 weeks gestation in Shenzhen, China. Maitre et al. suggested that the implementation of this model could result in earlier detection of CP (9). We therefore hypothesized that the implementation of the multidisciplinary assessment and care (MDAC) clinic would lead to an earlier diagnosis of NDI and CP, when compared to the conventional follow-up program of high-risk preterm infants.

## Methods

This retrospective observational study was approved by the University of Hong Kong–Shenzhen Hospital Institutional Ethics Committee [#(2021)058]. Written informed consent from the participants' legal guardian/next of kin was not required to participate in this study in accordance with the national legislation and the institutional requirements.

In Shenzhen, there are ~200,000 births annually in a population of 20 million. There are ~300 neonates admitted to the neonatal intensive care unit (NICU) at University of Hong Kong–Shenzhen Hospital annually. Our NICU provided family-centered care since 2013. Clinical and demographic information during hospital stay was collected in a prospective database, and the diagnosis of neonatal morbidities including neonatal sepsis, bronchopulmonary dysplasia, intraventricular hemorrhage, necrotizing enterocolitis, and retinopathy of prematurity followed that of international definitions (13). The data from all preterm infants, who were born at <29 weeks gestation from January 2015 to December 2019, survived and discharged from the NICU, and seen at the follow-up clinic in 2015–2020, were included in the study.

### High-Risk Infant Follow-Up Clinic in 2015–2017 (Pre-MDAC Epoch)

The high-risk infant follow-up clinic at the University of Hong Kong–Shenzhen Hospital routinely followed all neonates who were born in this hospital with medical complexity in the perinatal period including but not limited to prematurity, low birth weight (<2,500 g), small for gestational age, asphyxia, neonatal encephalopathy, neonatal stroke, congenital heart defects, or other anomalies. It was held twice weekly. Many children with less common risk factors for CP also received care in this clinic. The national guidelines recommended the follow-up of high-risk neonates at 6 weeks after discharge from the NICU and every 3–6 months thereafter. All visits included medical and neurological exams, needs assessment by neonatologists, and standardized testing by certified nurses (the Ages and Stages Questionnaire, v.3, ASQ-3; Zhuhai Ocean Educational Science & Technology Co., Ltd, Zhuhai, China, at 12 and 18 months). After assessment, patients with special needs are referred to subspecialty programs (audiology, ophthalmology, CP, and physical medicine) as appropriate or offered a follow-up appointment. There was no specific early CP diagnostic program.

### MDAC Clinic in 2018–2020 (MDAC Epoch)

In 2018, the follow-up of high-risk neonates for early diagnosis of CP was developed based on the model of the Nationwide Children's Hospital (8) and Novak et al. (11). An MDAC clinic that focused on following those preterm infants born at <29 weeks gestation was established, in addition to the visit at the high-risk infant follow-up clinic. The patient load at the MDAC clinic was lower than that of the high-risk infant follow-up clinic where a large population of high-risk infants were routinely monitored. This MDAC clinic was held monthly. The multidisciplinary team consisted of neonatologists; nursing specialists; and physical, occupational, and speech therapists. The team received intensive training by international specialists including developmental pediatricians, physiatrists, and therapists experienced in the assessment and care of neonates at risk for NDI and CP through regular workshops and clinical teachings. In this provider-organized MDAC clinic, eligible infants were seen at 6, 12, 18, and 24 months adjusted age.

Clinical information during hospital stay was documented by nursing specialists from the MDAC team. Social media (WeChat) was used to maintain contact with parents after discharge and understand the concerns and needs of families. Nursing specialists interviewed parents 1 day in advance of the MDAC clinic to record the progress of their child, including (1) general conditions such as seizures, feeding, sleep, and bladder and bowel habits, (2) results of vision and hearing tests, (3) the administration of ASQ-3 at 12–18 months, and (4) other parental concerns.

Following the guidelines of early diagnosis of CP (8), a unique model of “arena assessment” was implemented in this MDAC clinic. All team members attended a pre-assessment case conference to be familiar with every case based on up-to-date clinical information from nursing specialists. Each infant was examined by one team member with other members observing the examination in close proximity. The examiner completed and scored the Hammersmith Infant Neurological Examination (HINE) and the Alberta Infant Motor Scale (AIMS). The HINE is an easily performed, relatively brief, standardized, and scorable clinical neurological examination for infants between 2 and 24 months of age. HINE is accessible to all clinicians, with good inter-observer reliability even in less experienced staff. The use of the HINE optimality score and cut-off scores provides prognostic information on the severity of motor outcome. The HINE can further help to identify those infants needing specific rehabilitation programs (14). The AIMS is a unidimensional scale that aims to assess gross motor development of children aged up to 18 months by observing the spontaneous repertoire of children's skills detected through 58 items grouped under four postures: prone (21 items), supine (9 items), sitting (12 items), and standing (16 items) (15). Neonatal cranial ultrasonography, MRI brain, and ASQ-3 questionnaire were reviewed. Intraventricular hemorrhage was graded using the Papile classification (16). We used the classification system by Himmelmann et al. to categorize MRI findings (17). Infants were classified and diagnosed as having normal motor development, delayed motor development, high-risk of CP, CP, and NDI at the time of clinic attendance based upon the above assessments. Delayed motor development in preterm infants was defined by the mean value for the AIMS below 10th percentiles at 4 months and below 5th percentiles at 8 months (18). The HINE was completed using the standard proforma and scored from 0 to 78. An HINE score <73 (at 6, 9, or 12 months adjusted age) was considered at high risk of CP (11), whereas a score <59 and <65 at 6 and 12 months adjusted age, respectively, indicates CP (19). NDI was defined as one of neuromotor, neurocognitive, and neurosensory impairments at 12–18 months adjusted age. Neuromotor impairment included CP, AIMS <10th percentiles at 4 months, <5th percentiles at 8 months, an HINE score <59 at 6 months or <65 at 12 months adjusted age, or ASQ-3 scores in both gross and fine motor domains at the monitoring zone, or one that was below the cut-off threshold. Neurocognitive impairment was defined as ASQ-3 scores with ≥2 domains including communication, problem solving, and personal–social, were within the monitoring zone, or ≥1 domain below cut-off threshold (20). Neurosensory impairment included visual or hearing impairment requiring corrective measures. The category of NDI was determined by the most severe impairment in any domain. At the post-clinic conference, the MDAC team discussed and adjudicated the infants' neurodevelopmental state based on physical findings, ASQ-3, HINE and AIMS scores, and neuroimaging. As the team lead, the neonatologist then had a family-centered conference with the parents, discussed the findings, and provided anticipatory counseling and a care plan. Referrals were made for infants at high risk of CP or with CP to the department of physical medicine and rehabilitation for early interventions and therapy as appropriate. Infants who were diagnosed with NDI due to vision or hearing impairment were referred to the ophthalmology or otolaryngology departments for further management.

### Statistical Analysis

All analyses were conducted by using IBM Statistical Product and Service Solutions software Version 24 (SPSS Inc., Chicago, IL, USA). Continuous variables were summarized as the mean ± standard deviation and median with interquartile ranges (IQRs) for parametric and non-parametric distributions, respectively. The variables of two cohorts (pre-MDAC and MDAC epochs) were compared as well as those of infants diagnosed to have NDI and CP in the respective epoch. The parametric (Student's *t*) or non-parametric (Mann–Whitney *U*) tests were used to analyze variables accordingly. Comparisons of categorical variables were performed using the Pearson chi-square test or Fisher exact test. *P* < 0.05 was considered as statistically significant. Infants lost to follow-up in the study were not included in the analyses of the outcome variables.

## Results

From January 2015 to December 2019, 708 preterm neonates were admitted to our NICU with 73 (10%) born at <29 weeks' gestation. Thirteen (18%) of 73 neonates died during hospitalization. Among the 60 survivors, 49 (82%) had bronchopulmonary dysplasia, 28 (47%) had retinopathy of prematurity, 8 (13%) had severe necrotizing enterocolitis (stages II and III of Bell's classification), and 13 (22%) had major intraventricular hemorrhage (grades 3 and 4 of Papile staging). Table 1 shows the socio-demographic and perinatal–neonatal characteristics of all infants in the follow-up clinics. Chorioamnionitis, small for gestational age, longer hospital stay, prolonged invasive ventilation, and more frequent packed red blood cell transfusions were common among those with NDI compared with those without NDI (*P* < 0.05). Gestational age, birth weight, sex, delivery mode, Apgar score at 5 min of life, maternal ages, premature rupture of membrane, gestational diabetes mellitus, gestational hypertension, antenatal steroids, magnesium sulfate, surfactant therapy, lowest temperature within 12 h after birth, surfactant therapy, non-invasive ventilation days, neonatal sepsis, bronchopulmonary dysplasia, major intraventricular hemorrhage, severe necrotizing enterocolitis, all types of retinopathy of prematurity, discharged on home oxygen, discharged weight, corrected age of MRI, abnormal MRI findings, and periventricular leukomalacia were not different between infants with NDI and without NDI. Magnetic resonance imaging was performed in 48 (91%) infants, of whom 13 (27%) had abnormal brain findings including periventricular leukomalacia (*n* = 5) (Table 1).

**Table 1:** Socio-demographic and perinatal-neonatal characteristics between neurodevelopmental impairment (NDI) and non-NDI groups.

**Variables**	**Non-NDI (*n* = 29)**	**NDI (*n* = 24)**	***P***
Gestational age (weeks)	27.4 (26.6–28.1)	27.8 (26.4–28.2)	0.865
Birth weight (grams)	1,000 (920–1,105)	870 (735–1,100)	0.168
Male gender	14 (48%)	15 (63%)	0.300
Vaginal delivery	18 (62%)	13 (54%)	0.561
Apgar score at 5 min	8 (7–9)	8 (7–8)	0.531
Maternal age (years)	32 ± 4	34 ± 4	0.208
Premature rupture of membrane	3 (10%)	7 (29%)	0.081
Gestational diabetes mellitus	6 (21%)	3 (13%)	0.487
Gestational hypertension	2 (7%)	3 (13%)	0.649
Antenatal steroids	26 (90%)	22 (92%)	1.000
Magnesium sulfate	22 (76%)	13 (54%)	0.097
Maternal chorioamnionitis	3 (10%)	8 (33%)	**0.040**
SGA (birth weight <10th percentile)	0 (0%)	4 (17%)	**0.036**
Hospital length of stay (days)	76 (58–95)	91 (72–116)	**0.043**
Surfactant therapy (%)	15 (52%)	14 (58%)	0.630
Lowest temperature in 0–12 h (°C)	36.5 (35.8–36.7)	36.4 (36.0–36.7)	0.525
Duration of invasive ventilation (days)	0.04 (0–7)	6.75 (0.35–14.9	**0.028**
Duration of non-invasive ventilation (days)	51 ± 19	54 ± 23	0.568
Number of packed RBC transfusions	1 (0–3)	3 (2–4)	**0.016**
Neonatal sepsis	7 (24%)	7 (29%)	0.679
Bronchopulmonary dysplasia	25 (86%)	19 (79%)	0.715
Intraventricular hemorrhage (≥grade3)	5 (17%)	7 (29%)	0.302
Necrotic enterocolitis (≥stage II)	3 (10%)	5 (21%)	0.444
Retinopathy of prematurity	13 (45%)	15 (63%)	0.200
Discharged on home oxygen	1 (3%)	5 (21%)	0.080
Discharge weight (grams)	2,725 (2,260–2,995)	2,700 (2,390–3,363)	0.514
Corrected age of MRI (weeks)	38.5 (36.4–41.1)	39.7 (37.4–42.4)	0.192
Abnormal brain MRI	6/26 (23%)	7/22 (32%)	0.497
Periventricular leukomalacia	1 (3%)	4 (17%)	0.164

*SGA, small gestational age; RBC, red blood cell; MRI, magnetic resonance imaging; The bold values were used to indicate significance with P-values < 0.05*.

In the pre-MDAC epoch (2015–2017), 6 (20%) of 30 surviving infants lost to follow-up, whereas, 1 (3%) of 30 survivors lost to follow-up in the MDAC epoch (2018–2020) (*P* > 0.05). The remaining 53 infants were born at 27.3 ± 1.3 weeks gestation with birth weight 971 ± 215 g and 29 (55%) of male sex. Twenty-four (45%) infants had NDI. Five (9%) infants were diagnosed with CP, with 2 (4%) infants <12 months adjusted age diagnosed as at high risk for CP. Severe visual and hearing impairments were diagnosed in 4 (8%) and 2 (4%) surviving infants, respectively (Table 2).

**Table 2:** Neurodevelopmental outcomes during the pre-MDAC (2015–2017) and MDAC (2018–2020) epochs.

	**Pre-MDAC**	**MDAC**
	**(*n* = 24)**	**(*n* = 29)**
Total neurodevelopmental impairment (%)	12 (50%)	12 (41%)
Mild neurodevelopmental impairment (%)	5 (20%)	9 (32%)
Moderate to severe neurodevelopmental	7 (29%)	3 (10%)
impairment (%)		
Cerebral palsy (%)	3 (12%)	2 (7%)
High-risk of cerebral palsy (%)	—	2 (7%)
Visual impairment (%)	3 (12%)	1 (4%)
Hearing impairment (%)	1 (4%)	1 (4%)

*All P > 0.05*.

*Mild neurodevelopmental impairment: one of neuromotor impairment (GMFCS 2 or AIMS <10th percentile at 4 month, <5th percentile at 8 month or HINE <59 at 6 month, <65 at 12 month corrected age or both gross and fine motor domains of ASO-3 in monitoring zone, or one is below cut-off), neurocognitive impairment (≥2 domains pf ASQ-3 including communication, problem solving and personal-social, scores in monitoring zone, or one of domains below cut-off) or neurosensory impairment (visual or hearing deficits not requiring corrective measures)*.

*Moderate to severe neurodevelopmental impairment: a composite of neuromotor and neurocognitive and/or neurosensory impairment (GMFCS 3-5, or HINE <40 or both gross and fine motor domains of ASO-3 are below cut-off and ≥2 domains including communication, problem solving and personal-social below cut-off; and/or visual or hearing deficits requiring corrective measures)*.

*High-risk of cerebral palsy: HINE score <73 at 6, 9, or 12 months adjusted age, respectively*.

During the two epochs, ASQ-3 questionnaires were administered to 49 (92%) infants (Table 3) at the mean-adjusted age of 13.5 and 14.4 months for those infants without and with NDI, respectively. There were three un-validated completion of ASQ-3 questionnaires and one parental refusal in pre-MDAC and MDAC epochs, respectively. It was common for the scores of fine motor, personal–social, problem-solving, and communication domains to be below cut-off threshold among those infants with NDI, when compared with those without NDI (*P* < 0.05). Regarding the scores below the cut-off threshold of the gross motor domain, there was no difference between NDI and non-NDI infants (*P* = 0.072).

**Table 3:** Results of Ages and Stages Questionnaire (v.3) assessment in neurodevelopmental impairment (NDI) and non-NDI groups.

**Variables**	**Non-NDI (*n* = 28)**	**NDI (*n* = 21)**	***P***
Corrected age (month)	13.5 ± 7.5	14.4 ± 4.9	0.202
Gross motor below cut-off	0 (0%)	3 (14.3%)	0.072
Gross motor monitoring zone	2 (7.1%)	11 (52.4%)	**0.001**
Fine motor below cut-off	0 (0%)	4 (19.0%)	**0.028**
Fine motor monitoring zone	1 (3.6%)	4 (19.0%)	0.150
Communication below cut-off	0 (0%)	5 (23.8%)	**0.011**
Communication monitoring zone	1 (3.6%)	5 (23.8%)	0.072
Problem solving below cut-off	0 (0%)	4 (19.0%)	**0.028**
Problem solving monitoring zone	0 (0%)	2 (9.5%)	0.179
Personal-social below cut-off	0 (0%)	4 (19.0%)	**0.028**
Personal-social monitoring zone	1 (3.6%)	4 (19.0%)	0.150

*There was no validated data in Ages and Stages Questionnaire (v.3) of one child and 3 children in non-NDI and NDI groups, respectively. The bold values were used to indicate significance with P-values < 0.05*.

During the MDAC epoch, HINE and AIMS were implemented in MDAC clinic by the team. The mean adjusted age at the diagnosis of NDI and CP during the MDAC epoch was significantly lower than that during the pre-MDAC epoch [6 (5–12) vs. 14 (11–18) months, respectively, *P* = 0.02] (Table 4). There were no significant differences regarding demographic, clinical and neuroimaging findings, and main short-term morbidity between surviving infants in the pre-MDAC and MDAC epochs, although infants assessed in the MDAC epoch had a higher discharge weight (*P* = 0.045) (Table 4). The infants of the pre-MDAC epoch and a lower score in communication domain (*P* < 0.001) than infants assessed in the MDAC epoch (Table 4).

**Table 4:** Demographic characteristics, main morbidity, and Ages and Stages Questionnaire (v.3) (ASQ-3) scores of infants with neurodevelopmental impairment during pre-MDAC (2015–2017) and MDAC (2018–2020) epochs.

**Variables**	**Pre-MDAC (*n* = 12)**	**MDAC (*n* = 12)**	***P***
Gestational age (weeks)	28.0 (24.9–28.3)	27.1 (26.4–28.3)	0.887
Birth weight (grams)	880 (680–1,100)	850 (745–1,100)	0.843
Male gender	6 (50%)	9 (75%)	0.400
Vaginal delivery	7 (58%)	6 (50%)	0.682
Apgar score (5 min)	8 (7–8)	8 (8–9)	0.128
Maternal age (years)	33 ± 4	34 ± 4	0.389
Premature rupture of membrane	2 (17%)	5 (42%)	0.371
Gestational diabetes mellitus	1 (8%)	2 (17%)	1.000
Gestational hypertension	2 (17%)	1 (8%)	1.000
Antenatal steroids	11 (92%)	11 (92%)	1.000
Magnesium sulfate	5 (42%)	8 (67%)	0.219
Chorioamnionitis	3 (25%)	5 (42%)	0.667
SGA (birth weight <10th percentile)	2 (17%)	2 (17%)	1.000
Hospital length of stay (days)	104 ± 43	99 ± 31	0.912
Surfactant therapy (%)	8 (67%)	6 (50%)	0.408
Lowest temperature in 0–12 h (°C)	36.3 (35.5–36.6)	36.4 (36.3–36.8)	0.198
Duration of invasive ventilation (days)	8 (0–29)	7 (0.8–11)	0.799
Duration of non-invasive ventilation (days)	49 ± 15	60 ± 29	0.224
Number of packed RBC transfusions	4 (2–4)	3 (1–4)	0.347
Neonatal sepsis	5 (42%)	2 (17%)	0.371
Bronchopulmonary dysplasia	10 (83%)	9 (75%)	1.000
Intraventricular hemorrhage (≥grade3)	4 (33%)	7 (58%)	0.219
Necrotic enterocolitis of newborn (≥stage II)	3 (25%)	2 (17%)	1.000
Retinopathy of prematurity	9 (75%)	6 (50%)	0.400
Discharged on home oxygen	2 (17%)	3 (25%)	1.000
Discharge weight (grams)	2657 ± 382	3141 ± 741	**0.045**
Corrected age of MRI (weeks)	39 (37–42)	41 (38-42)	0.821
Abnormal brain MRI	4/10 (40%)	3/12 (25%)	0.652
Time of NDI and CP diagnosis (months)	14 (11–18)	6 (5–12)	**0.020**
ASQ-3 [*n* (%)]	10 (83%)	11 (92%)	1.000
Corrected age of ASQ-3 (months)	17.0 (11.3–22.5)	13.5 (11.2–17.1)	0.195
ASQ-3: Gross motor	29.2 ± 19.8	33.8 ± 16.4	0.543
ASQ-3: Fine motor	47.5 (20.0–50.0)	45.0 (35.0–57.5)	0.378
ASQ-3: Problem solving	30.0 (20.0–50.0)	40.0 (35.0–50.0)	0.242
ASQ-3: Personal-social	30.0 (12.5–40.0)	40.0 (25.0–50.0)	0.101
ASQ-3: Communication	24.2 ± 11.6	48.8 ± 10.1	**<0.001**
Hammersmith infant neurological examination score	—	48.5 ± 10	
Alberta infant motor scale score	—	15 (11–19)	

*SGA, small gestational age; RBC, red blood cell; MRI, magnetic resonance imaging. The bold values were used to indicate significance with P-values < 0.05*.

## Discussion

This is the first report describing the implementation of early CP and NDI diagnosis using a family-centered MDAC clinic in a Chinese setting. The incidence of moderate to severe NDI was 19% in this study, compared to 16–32% reported in a National Institute of Child Health and Human Development study (4). The incidence of severe NDI in survivors between 18 and 26 months adjusted age ranged from 3.5 to 14.9% (*n* = 2187) (2). A Swedish study incidence of severe NDI was 11% at 2.5 years of age for their more immature cohort born at <27 weeks of gestation (21). The rate of CP (9%) in this study was lower than that observed by Adams-Chapman et al. (vs. 12%), whereas that of severe visual impairment (8%) was higher and severe hearing impairment (4%) was (vs. 1 and 3%, respectively) (4). We believe that the difference could be related to the variation in definitions, reporting mechanisms, cohort characteristics and corrected ages at the time of assessment and diagnosis.

Epidemiological studies have shown that the origins of most CP are prior to labor.

Maternal chorioamnionitis is associated with an increased risk of CP in term infants (21). Among a case-control study reported a strong association between maternal chorioamnionitis and CP (odds ratio 4.1, 95% confidence intervals 1.6–10.1) (22). Other risk factors include prematurity and small for gestational age (23). Mechanical ventilation has been associated with increased risk of CP (24). Recent studies on white matter microstructure in extremely preterm infants (gestational age <27 weeks) found that the number of days on mechanical ventilation was an independent contributor to diffuse white matter injury, especially in the right external capsule (25), the occipital periventricular zone, and the centrum semiovale (26). Red blood cell transfusion has a negative impact on survival in extremely low-birth- weight infants. The number of transfusions affects later neurodevelopment (27). We observed similar findings in the current study with higher incidence of maternal chorioamnionitis, small for gestational age, packed cell transfusion, and longer days of mechanical ventilation and hospital stay in the NDI group, when compared to those variables of non-NDI group (Table 1).

The attendance rate at the MDAC Clinic (97%) was higher than the pre-MDAC clinic (81%) but did not reach statistical significance, probably due to our small sample. Some clinicians had different comfort levels with giving the diagnosis of high-risk for CP or CP in our new MDAC program. Acquiring experience and establishing confidence in administering AIMS and HINE was at times difficult. For all these reasons, a team-based system with mutual support was established so that providers could always discuss and rely on a more advanced or experienced observer for consultative assistance. A regular case review once a quarter and workshops twice per year were held with the clinical experts in neonatal follow-up and physical medicine and rehabilitation (MJW) for quality assurance. For diagnosis, if the clinician did not feel comfortable or assessed that the family was not ready (emotional state, lack of support system, other parent not present, child acutely ill, or crying at visit) to receive a diagnosis, parents were counseled regarding delayed neurodevelopment and reassessment was arranged within 1–3 months to ensure early communication of diagnosis, initiate early intervention, and parental support and counseling.

The guidelines by Novak et al. recommend that all high-risk infants should have MRI brain, HINE and AIMS performed at >5 months' corrected age to allow for the early diagnosis of CP (12). All infants, who did not have MRI brain at term corrected age, had at least three cranial ultrasound examinations during the stay in our NICU. Cranial ultrasound examinations have been shown to have a similar specificity to MRI for the diagnosis of CP but are less sensitive at detecting white matter changes at term (28). Cognitive impairment is significantly correlated with birth-weight and gestation age (29). Cognitive impairment is commonly measured using the cognitive scale or mental developmental index of the Bayley Scales of Infant Development or cognitive domains of ASQ-3. The American Academy of Pediatrics recommended the use of a parent-reported developmental screening tool, the ASQ-3, which had 75% sensitivity and 81% specificity when compared to the Bayley Scale of Infant Development-II (30). The ASQ-3 was performed in 92% of infants in this study (Table 3). In this study, low cognitive functions were significantly associated with positive ASQ-3 results in communication, problem solving and personal-social skills at 14 months corrected age, but not with gross motor skills. The correlation between communication and gross motor domains was the lowest, while the correlation between communication and problem solving was the highest, which is similar to the domain correlations reported previously by Agarwal et al. (31). The earliest sign of NDI is most likely manifested by motor impairment in the first year of life, in the form of CP or suspected CP, as language developmental abnormality may not be evident, except for feeding difficulties. Postnatal under-nutrition and faltering growth are common and associated with adverse cortical development in the neonatal period (32) and long-term neurodevelopmental outcomes (33). In a group of 613 babies born <33 weeks' gestational age, assessed at 18 months' corrected age, Belfort et al. found that every-one z-score improvement in weight gain and body mass index between 1 week of age and term-corrected age was associated with an increase in Bayley II mental developmental indices of 2.4 and 1.7 points and psychomotor developmental indices of 2.7 and 2.5 points, respectively (34). Interestingly, we found that significantly higher body weight at discharge in those infants with NDI during the MDAC epoch, when compared with that during the pre-MDAC epoch (Table 4). Further, these infants of the pre-MDAC epoch had a lower cut-off score of ASQ-Communication (*P* < 0.001) but not with other domains, which was similar to that in the study by Belfort et al. (35).

Compared with that during the pre-MDAC epoch, infants followed up in the MDAC program had CP diagnosed earlier (mean age 6 months) with mean cut-off of HINE scores of 48.5 ± 10 and mean AIMS scores of 15 (11–19) (<10th percentile). The age at diagnosis of CP in pre-MDAC epoch was 14 (range 11–18) months, which was similar to that of some low- and middle-income countries (5–7). The inclusion of a “precision” CP diagnosis program may contribute to the early diagnosis of CP during the MDAC epoch. Indeed, the identification of CP can be challenging in low- and middle-income countries because of the lack of resources and tools. However, HINE and AIMS are user-friendly. The AIMS is a unidimensional scale by observing the spontaneous repertoire of children's skills. Multilingual (English, Spanish, and French) versions of the HINE video and forms are available online at no cost (14), whereas other scales are costly or have lengthy certifications or proprietary forms. International guidelines for early detection of CP recommended using HINE, the most predictive neurological examination for CP, in the first year of life when the General Movements Assessment cannot be performed at 3–4 months of age or in countries where MRI is not available or affordable (11). Our findings and experience may benefit low- and middle-income countries.

There is evidence that the brain development and refinement of the motor system continue postnatally, driven by motor cortex activity (34). Early active movement and interventions are essential because infants who do not actively use their motor cortex risk lose cortical connections and dedicated function. CP-specific early intervention maximizes neuroplasticity and minimizes deleterious modifications to muscle and bone growth and development (36). Early diagnosis and early intervention are important to optimize infant motor and cognitive plasticity, prevent secondary complications, and enhance caregiver well-being. In this study, the decrease in age at the diagnosis of CP could be because of the creation of the MDAC clinic or because of the incorporation of the HINE and the AIMS (or both). However, one of the original aims for the creation of the MDAC clinic was to reduce the rate of loss to follow-up, which was not significant due to small sample size.

There are several limitations of this study. Firstly, this is a retrospective study of a small cohort of patients in a single center that precluded examining the effects of confounding variables. Secondly, we did not routinely use the General Movements Assessment prior to discharge from the NICU, which may help identify infants meeting the “infant-attributable risk” pathway of the international guidelines (11). Thirdly, we did not have the gold-standard developmental assessment in this age range, the Bayley Scale of Infant Development, because the translated and updated versions of the Bayley Scale of Infant Development are not available in China for various reasons including but not limited to logistics, cost, and copyright. Indeed, it is very challenging to establish an MDAC clinic in low- and middle-income countries. While it would be better to use these tests, we used assessment tools (HINE and AIMS) that are free and user-friendly.

In conclusion, in this pilot observational cohort study of critically ill preterm neonates with gestation age <29 weeks, a multidisciplinary “arena assessment” model with HINE, AIMS, ASQ-3, and MRI brain scan as objective measures is feasible and effective for an early diagnosis of NDI and CP in China. If other centers and studies confirm our findings and experience, early diagnosis of motor impairment may facilitate earlier identification of NDI in this population, especially in the low- and middle-income countries. A formal diagnosis of CP or high-risk of CP is essential for families to access necessary intervention and support in China.

## Data Availability Statement

The raw data supporting the conclusions of this article will be made available by the authors, without undue reservation.

## Ethics Statement

The studies involving human participants were reviewed and approved by University of Hong Kong-Shenzhen Hospital Institutional Ethics Committee [#(2021)058]. Written informed consent from the participants' legal guardian/next of kin was not required to participate in this study in accordance with the national legislation and the institutional requirements.

## Author Contributions

H-BH conceptualized and designed the study, drafted the initial manuscript, and revised the manuscript. P-YC conceptualized and designed the study, supervised clinical information/data collection, critically reviewed, and revised the manuscript. MW set up the MDAC clinic, implemented training of AIMS and HINE to staff, supervised clinical assessment, and revised the manuscript. Q-SZ conceptualized clinical information/data collection and critically reviewed the manuscript for important intellectual content. MH implemented HINE training and critically reviewed the manuscript for important intellectual content. C-BC set up the high-risk infants follow-up clinic and supervised clinical information/data collection. FL and X-QW were involved in the care and assessment of the follow-up patient, collected clinical information/data, and critically reviewed the manuscript for important intellectual content. All authors approved the final manuscript as submitted and agreed to be accountable for all aspects of the work.

## Funding

This study was funded by Shenzhen SanMing Project of Medicine (SZSM 201911016).

## Conflict of Interest

The authors declare that the research was conducted in the absence of any commercial or financial relationships that could be construed as a potential conflict of interest.

## Publisher's Note

All claims expressed in this article are solely those of the authors and do not necessarily represent those of their affiliated organizations, or those of the publisher, the editors and the reviewers. Any product that may be evaluated in this article, or claim that may be made by its manufacturer, is not guaranteed or endorsed by the publisher.
